# Counting what counts: assessing quality of life and its social determinants among nursing home residents with dementia

**DOI:** 10.1186/s12877-024-04710-1

**Published:** 2024-02-21

**Authors:** Matthias Hoben, Emily Dymchuk, Malcolm B. Doupe, Janice Keefe, Katie Aubrecht, Christine Kelly, Kelli Stajduhar, Sube Banerjee, Hannah M. O’Rourke, Stephanie Chamberlain, Anna Beeber, Jordana Salma, Pamela Jarrett, Amit Arya, Kyle Corbett, Rashmi Devkota, Melissa Ristau, Shovana Shrestha, Carole A. Estabrooks

**Affiliations:** 1 School of Health Policy and Management, Faculty of Health, York University, Room 301E Stong College, 4700 Keele StreetON, Toronto, M3J 1P3 Canada; 2 Faculty of Nursing, College of Health Sciences, University of Alberta, Edmonton, AB Canada; 3 Rady Faculty of Health Sciences, Max Rady College of Medicine, University of Manitoba, Winnipeg, MB Canada; 4 Nova Scotia Centre on Aging, Mount Saint Vincent University, Halifax, Canada; 5 Department of Sociology, Faculty of Arts, St. Francis Xavier University, Antigonish, NS Canada; 6 School of Nursing, Faculty of Human & Social Development, University of Victoria, Victoria, BC Canada; 7 Faculty of Medicine and Health Sciences, University of Nottingham, Nottingham, UK; 8 School of Nursing, Johns Hopkins University, Baltimore, MD USA; 9 Faculty of Medicine, Dalhousie University, Horizon Health Network, Saint John, New Brunswick Canada; 10 Freeman Centre for the Advancement of Palliative Care, North York General Hospital, Toronto, ON Canada; 11 Specialist Palliative Care in Long-Term Care Outreach Team, Kensington Gardens Long-Term Care, Kensington Health, Toronto, ON Canada; 12 Division of Palliative Care, Department of Family & Community Medicine, University of Toronto, Toronto, ON Canada; 13 Division of Palliative Care, Department of Family Medicine, McMaster University, Hamilton, ON Canada; 14 Dr. Gerald Zetter Care Centre, The Good Samaritan Society, Edmonton, AB Canada

**Keywords:** Quality of life, Dementia, Nursing homes, Social determinants of health

## Abstract

**Background:**

Maximizing quality of life (QoL) is a major goal of care for people with dementia in nursing homes (NHs). Social determinants are critical for residents' QoL. However, similar to the United States and other countries, most Canadian NHs routinely monitor and publicly report quality of care, but not resident QoL and its social determinants. Therefore, we lack robust, quantitative studies evaluating the association of multiple intersecting social determinants with NH residents’ QoL. The goal of this study is to address this critical knowledge gap.

**Methods:**

We will recruit a random sample of 80 NHs from 5 Canadian provinces (Alberta, British Columbia, Manitoba, Nova Scotia, Ontario). We will stratify facilities by urban/rural location, for-profit/not-for-profit ownership, and size (above/below median number of beds among urban versus rural facilities in each province). In video-based structured interviews with care staff, we will complete QoL assessments for each of ~ 4,320 residents, using the DEMQOL-CH, a validated, feasible tool for this purpose. We will also assess resident’s social determinants of QoL, using items from validated Canadian population surveys. Health and quality of care data will come from routinely collected Resident Assessment Instrument – Minimum Data Set 2.0 records. Knowledge users (health system decision makers, Alzheimer Societies, NH managers, care staff, people with dementia and their family/friend caregivers) have been involved in the design of this study, and we will partner with them throughout the study. We will share and discuss study findings with knowledge users in web-based summits with embedded focus groups. This will provide much needed data on knowledge users' interpretations, usefulness and intended use of data on NH residents’ QoL and its health and social determinants.

**Discussion:**

This large-scale, robust, quantitative study will address a major knowledge gap by assessing QoL and multiple intersecting social determinants of QoL among NH residents with dementia. We will also generate evidence on clusters of intersecting social determinants of QoL. This study will be a prerequisite for future studies to investigate in depth the mechanisms leading to QoL inequities in LTC, longitudinal studies to identify trajectories in QoL, and robust intervention studies aiming to reduce these inequities.

**Supplementary Information:**

The online version contains supplementary material available at 10.1186/s12877-024-04710-1.

## Background

Improving quality of life (QoL) – a person’s physical, emotional and social well-being – is a priority goal of care for people living with dementia [1–4]. In 2019, 55 million people worldwide were living with dementia, a number expected to increase to 139 million by 2050 [5]. Dementia is a progressive and irreversible syndrome, caused by a range of neurodegenerative, vascular, and other disorders that contribute to decline in cognitive and functional abilities, neuropsychiatric symptoms, and lead to death [6, 7]. Yet, even with profound impairment, people living with dementia can have good QoL with appropriate supports [8]. While communication difficulties, behavioral disturbances, and functional limitations can negatively affect QoL [9], they do not have to [6, 10]. People living with dementia, even those with severe cognitive and physical impairment, often have good QoL [11, 12].

Because appropriate supports are not consistently available, having dementia [13] is a major risk factor for poor QoL, especially for nursing home (NH) residents [14]. Of the 250,000 Canadian NH residents [15], 69% have a diagnosis of dementia, and 89% have some form of cognitive impairment [16]. NH policy prioritizes physical safety and security over QoL [17]. Current research and public reporting in Canada are largely based on the Resident Assessment Instrument – Minimum Data Set 2.0 (RAI) [18], which does not measure QoL [19]. The RAI provides objective indicators of care quality, such as inappropriate antipsychotics use, physical restraint use, pain, and depressive symptoms [20, 21]. These burdensome conditions and treatments negatively affect QoL [22, 23], but their absence does not guarantee good QoL [24]. RAI quality indicators do not include key aspects of QoL, such as social relationships, sense of purpose, or a resident’s feelings and worries [19, 24]. Measuring QoL directly, rather than relying on dementia severity as a poor proxy, is essential to accurately monitor a person’s well-being, and to enable design and evaluation of specific interventions to improve their QoL.

International research on NH residents’ QoL and its social determinants is limited. Social determinants are the non-medical conditions that shape people’s daily lives and affect their health and QoL [25]. They are the major drivers of health inequities, the unfair and avoidable differences in health and QoL among populations [26]. Intersecting identities, such as class, age, gender, race and ethnicity, disability, or religion combine to create various modes of privilege and discrimination, all of which can affect QoL [27, 28]. A recent systematic review and meta-analysis assessed factors associated with QoL in people with dementia [10]. Studies in NHs and assisted living suggested that lack of person-centered care and longer duration of stay decreased QoL. These studies did not assess any social determinants of QoL other than age and gender (both found to be unrelated to QoL) [10]. Studies in non-institutional settings found that being white, having good social relationships, being more socially engaged, being cared for by a spouse, being married, and having religious/spiritual beliefs were associated with better QoL [10]. Living alone decreased QoL [10]. US research suggests that racialized^1^ NH residents or those who lived in a NH with high proportions of racialized residents had poorer QoL [29–33]. US research has also examined social determinants associated with inequities in quality of care and healthcare access [34], but we lack research assessing the association of social determinants other than racialization on the QoL of NH residents, especially those with dementia. Also, US research may not apply to Canada or other countries because NH systems [35], population structures [36], and historic, legal, political, and economic contexts differ.

A recent Canadian study found that non-English speaking NH residents had an increased risk of hospitalization [37]. Our work in the Canadian province of Alberta found lower QoL among racialized NH residents [38]. Canadian qualitative studies illustrate barriers encountered by racialized older adults in accessing healthcare services [39], and the multiple challenges they face in maintaining good QoL in assisted living [40]. The only Canadian study assessing QoL in NHs across provinces was conducted over a decade ago [41, 42]. However, this study did not assess social determinants of QoL, such as racialization, social supports, or financial situation. It also only included residents with no or low cognitive impairment who could self-report, excluding those whose QoL is probably most at risk. No cross-provincial comparisons were reported, even though NH care is regulated provincially in Canada and outcomes vary substantially across provinces [17, 43, 44]. A convenience sample of residents and NHs limited generalizability of findings. We lack large-scale quantitative evaluations of NH residents’ QoL and its social determinants (beyond racialization). We especially lack Canadian data, and data on how factors may intersect to influence QoL [10].

Our study aims to compare QoL among subgroups of older adults living with dementia in NHs, who experience multiple intersecting vulnerabilities – including residents at advanced stages of dementia who cannot self-report and who have no family member or friend to provide a proxy assessment. Three specific research objectives will address these pressing issues:

1. Assess QoL and associated social determinants in a representative sample of NH residents in five Canadian provinces;
2. Identify clusters of intersecting social determinants that perpetuate and reinforce QoL inequities among NH residents;
3. Evaluate, through focus groups and knowledge users’ interpretations, the perceived usefulness and intended uses of data on NH residents’ QoL and its social determinants.

## Methods and design

In this 3-year (April 2022 to March 2025) explanatory sequential mixed methods study [45], we will use an integrated knowledge translation (iKT) approach [46] to co-produce knowledge with knowledge users in all project phases. We will first collect quantitative data on nursing residents’ QoL and its social determinants from a representative sample of NHs in the Canadian provinces of Alberta (AB), British Columbia (BC), Manitoba (MB), Nova Scotia (NS), and Ontario (ON). After obtaining provincial ethics approvals and operational approvals (details in Objective 1: Assess QoL and its social determinants section, April to December 2022), we started with the recruitment of facilities (current phase of the project). Data collection started in November 2022 in the provinces and regions we received ethics/operational approvals first, and is scheduled to be completed in April 2024. We will then assess QoL and its health and social determinants (objective 1), as well as different clusters of intersecting factors in those with the highest and lowest QoL (objective 2). Finally, we will prepare feedback reports and conduct feedback webinars with embedded focus groups with knowledge users (objective 3) to help us interpret our findings, discuss how results can be used to enhance care practices, and identify strategies to maximize QoL and minimize QoL inequities among NH residents with dementia.

### Theoretical framing

**Fig. 1:**
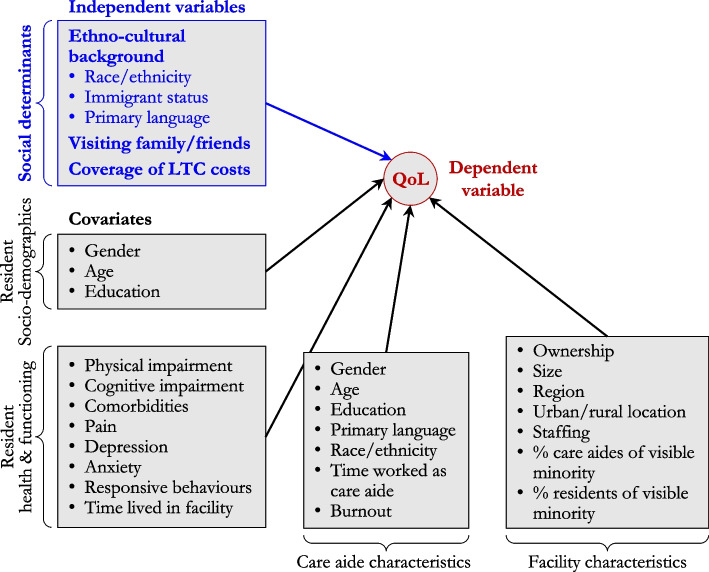
Social determinants and other factors (covariates) associated with nursing home residents’ QoL

Our theoretical framework integrates 4 perspectives. 1) Social determinants of health – the idea that living conditions primarily shape the health and well-being of individuals, not just medical treatments or lifestyle choices [25]. 2) A health equity framework [47] that outlines important social factors supporting or impeding health equities. 3) Intersectionality – an analytical framework to understand how intersecting, overlapping social identities combine to create different modes of discrimination and privilege [48, 49]. 4) The Wilson & Cleary QoL framework [50] adapted for NHs [51], which guided our selection of social determinants and model covariates (Fig. 1). In alignment with our iKT approach, this study will be guided by a panel of 33 knowledge users, including 4 older adults in need of care and their family/friend caregivers, 7 representatives of advocacy organizations (CanAge, provincial Alzheimer Societies, Canadian Society of Palliative Care Physicians), 9 persons working in NHs (care aides, regulated nurses, managers), and 13 health system decision makers. These knowledge users participated in the conceptualization of this study and they will contribute to each step of the research process by sharing their lived experiences, guiding our priorities, and helping us contextualize our findings.

### Objective 1: Assess QoL and its social determinants

In this observational survey study, we will use video calls to collect data on residents’ QoL and its social determinants from care staff who know residents well. We will obtain residents’ most recent, de-identified RAI assessment (completed by care staff in their routine practice) to assess resident health and function covariates. We will complete a survey with an administrator in each participating NH to assess home-level covariates. We will assign a random study identifier to each resident, so that we will not have to identify residents at any time. Therefore, the ethics boards who approved this study waived the requirement to obtain resident consent (Ethics approval and consent to participate section) .

#### Setting and sample

NHs will be eligible if they are publicly subsidized and licensed by the respective health authority, located in one of the participating health regions or zones, and have 15 beds or more. We will exclude congregate care settings for older adults, other than NHs, such as assisted/supportive/retirement living. In AB, all five Health Zones will participate (170 eligible homes, 15,191 beds [52]). In BC, 4 of 5 Health Regions will participate (260 eligible homes, 27,524 beds [53]). The BC Northern Health Region was excluded because it only has 15 eligible NHs, many of which serve primarily Indigenous populations, and our study team has not established the close, long-term relationships with Indigenous communities that are required to ethically conduct research including these populations. In MB, only the Winnipeg Regional Health Authority will participate (39 eligible homes, 5,640 beds [54]), since only homes in this region routinely collect the RAI data required for our analysis. In NS, all 4 health zones will participate (86 eligible homes, 6,862 beds). Ontario has a total of 617 eligible homes (79,197 beds) [55] and the province is not divided into health regions.

We aim to recruit 10% of all eligible NHs in each of AB (*N* = 17), BC (*N* = 26), Winnipeg (*N* = 4), and NS (*N* = 9). Ontario, Canada's largest province, has over 600 NHs, so recruiting a 10% sample is not feasible with the available resources. Therefore, we will recruit 24 homes in Ontario (slightly less than a 5% sample) for a total of 80 homes. We will randomly select sites, stratified by health region (geographic region in ON), urban/rural location, for-profit/not-for-profit status, and large/small size (using median number of beds for urban versus rural sites in each region as cut point). We chose these strata given their association with quality of care and QoL [56–59]. We aim to collect data from all eligible residents at each site. Residents will be included if they have cognitive impairment (a RAI Cognitive Performance Scale [CPS] score of 1 or higher; > 90% of all residents) and have been living in the home for at least 3 months, to ensure that care staff know residents well enough to assess their QoL and social determinants. Residents will be excluded if we cannot identify a suitable care aide. Care aides will be selected who have 1) cared for the resident on at least 4 days during the week before the QoL assessment (the DEMQOL-CH, our QoL assessment tool, assesses residents’ feelings/worries in the last 7 days); 2) worked morning and evening shifts (to ensure awareness of changes in residents’ feelings/worries that depend on time of the day); and 3) worked in the home for at least 3 months (to ensure familiarity with the resident over a longer time).

#### Sample Size

In our feasibility work [60], regression models on resident QoL suggested a medium effect size (f^2^ = 0.14) and a within-home intra-cluster correlation (ICC) of 0.211. QoL assessments of multiple residents provided by the same care aide were independent of the assessor (no significant clustering within care aides). To detect this medium effect, a linear multiple regression model with total DEMQOL-CH score as dependent variable, 30 covariates, α = 0.05, and power = 0.8 would require a sample size of 199 residents. Considering the nested structure of our data, we multiplied the required sample size by a variance inflation factor (VIF) [61]. VIF = 1 + (m-1)*ICC with m being cluster size (~ 50 participating residents/home). Our required sample size is 199*11.339 = 2,257 residents. Our feasibility study [60] found that QoL data could be collected from ~ 50% of the residents in participating homes. This corresponds to ~ 4,320 of the ~ 8,640 residents in our 80 NHs. This sample size is more than large enough to account for attrition, smaller effect sizes, and subgroup analyses.

#### Dependent variable – QoL

Systematic reviews [62–67] identified at least 22 tools to measure QoL for people with dementia. We chose the DEMQOL-CH (Additional file 1) [60, 68] because: 1) It is dementia-specific, assessing residents’ feelings and worries. Generic measures of QoL (e.g. EQ5D [69], SF-12 [70]) often work poorly for people with dementia. 2) It is among the most rigorously developed and validated dementia-specific QoL tools, with psychometric properties at least as good as those of other available tools [64, 71–77]. 3) It was developed and validated for completion by care aides [68]. 4) In our recent study [60] we found that this approach is highly feasible and robust (details below).

The DEMQOL-CH was developed to ensure that people with severe dementia can be included in evaluations. For those in NHs, the choice of proxy is family/friends or care staff. Unfortunately, many residents in Canadian NHs do not have family/friend proxies (up to 35% in some homes) [78]. Mixing family/friend and care staff proxy reports of QoL compromises comparability, because family/friend and care staff proxy reports differ systematically [67]. Family/friend ratings of resident QoL are not by default more accurate than care staff ratings. If a single measure is needed across severities of cognitive impairment and availability of family support (as in our study), staff proxy assessments are the only consistent source available to assess QoL.

Team member Banerjee and colleagues developed and validated the DEMQOL-CH [68]. It is based on the DEMQOL-Proxy [71, 72], an extensively validated tool [64, 65] that uses reports by a family/friend proxy to assess QoL in people with all levels of dementia severity. The 31 DEMQOL-CH items are rated on a 4-point scale (1 = *Not At All* to 4 = *A Lot*) and summed to create an overall score (possible range: 31–124). The DEMQOL-CH correlates well with Dementia Care Mapping, an established, robust, observation-based but highly time-consuming method to assess residents’ QoL [79]. Well-known predictors of QoL associate with DEMQOL-CH scores, internal consistency reliability is excellent (0.9), and test–retest reliability is acceptable (0.72) [68]. Inter-rater reliability was borderline-acceptable in the UK study (0.4) [68]. Our Canadian study [60] confirmed the excellent internal consistency reliability (0.83). We found much-improved inter-rater reliability (0.74) by asking administrators to select 2 care staff members for assessments who both knew the resident well. Care aides could complete the DEMQOL-CH within 5 min, and care staff and managers rated use of the DEMQOL-CH as highly feasible, acceptable, and valuable.

#### Independent variables – social determinants

For this first quantitative, large-scale evaluation of multiple intersecting social determinants of NH resident QoL in Canada, we will prioritize a focused set of social determinants that our knowledge users deemed highest priority. Data on these can be easily collected from residents and families by care teams, using a survey that includes items from validated Statistics Canada population surveys [80, 81] (Additional file 2). As outlined above, racialization is strongly associated with poor QoL [29–33]. We will also include a person’s immigrant status and primary language as variables. Visiting family/friend caregivers are essential to timely and appropriate care [82, 83]. They have critical roles as advocates [84] and legal decision makers for residents unable to clearly communicate their wishes or needs because of cognitive impairment [82, 83, 85]. We will include an item asking care staff and NH key contacts whether the resident is regularly visited by family or friends. Finally, a person’s socioeconomic situation is a strong predictor of their QoL. For example, recent immigrants face challenges accessing low-income supports, leading to longer wait times for NH placement [86]. While we cannot access a resident’s financial data, key contacts in participating homes will know whether a resident’s NH costs are fully covered publicly. In most Canadian provinces, NH costs are shared between authorities and care recipients unless the private share would cause the resident and their family serious financial hardship. Full public coverage of NH costs indicates a resident’s low income.

#### Resident covariates

These covariates will primarily be drawn from the RAI-MDS 2.0, a valid, reliable standard assessment of residents’ characteristics [18, 87]. Its 450 items capture physical and cognitive functioning and multiple symptoms. It is routinely administered at admission and quarterly in AB, BC, MB, and ON. In NS, a comparable version (RAI Long Term Care Facilities, LTCF) is collected [88, 89]. We will adjust our models for age, gender, education, physical and cognitive functioning, co-morbid conditions, pain, depression, anxiety, responsive behaviours, and length of stay [10]. Our team has established processes for obtaining de-identified RAI records from residents in participating NHs. Via date and resident identifiers, we can link RAI records collected in our survey time-period with resident QoL and social determinants data.

#### Care aide covariates

These covariates will be from a care aide demographic survey (Additional file 3) that we will complete with each participating care aide after the DEMQOL-CH assessment. The items are from a well-validated survey that our team has used extensively with care aides for decades [90]. We will include care aides’ age, gender, education, race/ethnicity, and primary language to control for possible care aide characteristics that may influence resident QoL or a care aide’s rating of resident QoL. Because care aides who are more distressed tend to rate residents’ QoL lower [67], we will also control for care aide burnout, using the well-validated 9-item Maslach Burnout Inventory [91–93].

#### NH covariates

Covariates will be from a NH survey (Additional file 4), completed with a key contact in each home. Items are based on a well-validated NH survey, used previously by our team [90]. We will include variables thought to be associated with NH residents’ QoL [10, 29–31, 33, 42, 56, 94, 95]: ownership model (public not-for-profit, voluntary not-for-profit, private for-profit), small vs large size based on the median bed size among urban vs rural homes in the respective region, health region (to account for regional differences in structures, regulations, and policies that may influence QoL), urban/rural location, staffing (care aide, licensed practical nurse, and registered nurse care hours per resident day based on a validated staffing measure [96]), and proportion of care aides and residents who are racialized, immigrants, or who speak English as an additional language.

#### Data collection

We will recruit NHs and work with a key contact in each site to identify eligible residents and care aides. Sites will receive a stipend of $500 for participation. Key contacts who help organize data collection will receive a $50 coffee gift card, and care aides completing the DEMQOL-CH will receive coffee gift cards ($5 for up to 5 residents assessed, increasing by $5 for every additional 1–5 residents assessed). Key contacts will complete a NH survey via structured phone or video interviews. Care aides will be interviewed via video or phone. De-identified RAI assessments (those completed within 8 weeks of the QoL assessment) will be obtained from the regional health authorities.

#### Data analyses

As per REporting of studies Conducted using Observational Routinely-collected Data (RECORD) [97] guidelines, we will conduct basic analyses to ensure that data are of high quality and comparable across jurisdictions. Our team has extensive cleaning protocols to ensure integrity and comparability of NH data. Analyses will apply multiple imputations as per best practices for managing missing data [98]. We will comprehensively describe resident QoL and other health and social characteristics, using summary statistics. We will stratify descriptive analyses of QoL scores by sampling strata (region, size, ownership, urban/rural location) and by resident (e.g., age category, gender, racialization/immigration status/language) and NH social determinants (e.g., above median proportion of racialized residents). We will then use hierarchical generalized linear mixed models [99] (Fig. 1) with QoL (DEMQOL-CH score) as the dependent variable to assess whether social determinants (independent variables) are associated with QoL. We will adjust all models for resident, care aide, and NH covariates. Models will also include a random home-level intercept to account for dependencies of residents living in the same home. We will account for regional differences by including region as a covariate in our models.

### Objective 2: Identify clusters of intersection social determinants of QoL

We will use survey data collected in objective 1 to 1) identify inequities in QoL and 2) assess if and how clusters (latent classes) of intersecting social determinants differ between residents with high and low QoL, using multi-level covariate-adjusted latent class analyses (LCAs) [100]. LCAs are widely used to identify subgroups in a population that are characterized by intersection of particular characteristics (in our case: clusters of intersecting social determinants) [101]. QoL inequities are unfair and avoidable differences in QoL [26]. Therefore, it is important to compare subsamples of residents with especially high and low QoL scores. Intersectionality means that social determinants such as racialization, class, or gender are interconnected, and embedded within systems and discourses of power that perpetuate structural inequities [48, 49]. Rather than modelling social determinants individually (or interactions of 2 social determinants) as in our regression models (objective 1), LCAs will allow us to identify which combinations (latent classes) of social determinants are present among residents with especially high and low QoL and which profiles are more common.

#### Sample and sample size

Analyses will include the 25% of residents (*n* = 1,080) in our sample with lowest QoL (25th percentile of DEMQOL-CH scores) and the 25% of residents with highest QoL (75th percentile of DEMQOL-CH scores). A simulation study of sample sizes required for multi-level LCA [102] suggests a minimum of 20 homes with at least 10 residents per home. Our total study sample will include ~ 50 residents in each of 80 homes. Therefore, our 25% subsamples will include ~ 12.5 residents per home, comfortably meeting sample size requirements. For sensitivity analysis and to mitigate any sample size issues, we will split the total sample based on the median DEMQOL-CH score and replicate our analyses. This will give us a total sample size of 2,160 residents in each of the 2 subsamples (high/low QoL) and ~ 25 residents per home.

#### Model variables

Models will include our main social determinants plus gender as latent class indicators. We will adjust the models for resident and care aide (level 1) and NH (level 2) covariates that are statistically significantly associated with resident QoL in our regression models (objective 1). Level 1 covariates predict an individual’s overall probability of being in a certain latent class. Level 2 covariates predict a NH’s probability that an individual will be in a certain latent class.

#### Data analyses

We will specify 2-level LCA models, adjusted for level 1 and level 2 covariates. Level 1 random class means are modelled as continuous level 2 latent variables that vary across homes (equivalent to a home-level random intercept) [100]. As in objective 1, we will include region as a model covariate rather than a random intercept. We will run separate series of LCA models in each of our 2 subsamples. In collaboration with our knowledge user experts and guided by our theoretical framework, we will pre-specify the expected number of classes, given the nature of social determinants included. We will then run models with 1, 2, and 3 more classes and with 1, 2, and 3 fewer classes than the number pre-specified [101]. We will compare the fit of each model to that of its predecessor using a significance test and model fit criteria [101]. We will select the final model based on theoretical and statistical considerations.

### Objective 3: Evaluate knowledge user’s interpretations, usefulness, and intended use of data

Using a qualitative descriptive approach [103], we will share and discuss findings with our knowledge users to understand their perspectives on how social determinants are related to QoL. We will ask participants how useful they find such data, and if and how they intend to use it in their practice. We will discuss with relevant partners how such data may be used to develop and evaluate future NH policy. Our knowledge users are highly committed to prioritizing and improving QoL in their jurisdictions and care settings, and our iKT approach will expedite their uptake of our study findings. This study is an essential precursor to using these data in future audit and feedback work to improve resident QoL. We will also discuss existing promising practices and potential new strategies to maximize residents’ QoL and minimize QoL inequities, which could be further developed and evaluated in future studies. Our approach uses: 1) systematic feedback of study findings to participating care teams and other knowledge users, in an effective, feasible, theory-based feedback approach previously tested [104–109], 2) interactive, facilitated discussions of our findings with knowledge users in recorded video conference-based summits, and 3) focus groups of 5–6 knowledge users each, who can speak to experiences of immigrants, racialized, and low income older adults living with dementia.

#### Feedback reports to share findings with knowledge users

For each NH and health region, we will generate a tailored feedback report, structured and designed like the feedback reports our team has successfully generated, disseminated and discussed with knowledge users for decades [104–109]. We will develop reports in collaboration with our knowledge users for wider implementation. Each feedback report will include the QoL findings (rating of each DEMQOL-CH item and overall DEMQOL-CH score) for each resident in that facility that we collected QoL data for. We will also report the facility's overall (average) DEMQOL-CH score and compare it to the rest of the (de-identified) sample (by region, province and overall). Each facility's unique report will only be shared with that facility. For knowledge users who are not part of any of the participating facilities, we will generate overall summary reports.

#### Sample – feedback summit participants

We will invite participating care teams, our study's knowledge users, and additional knowledge users to web-based feedback summits (3 summits to maximize numbers of attendees, 20–30 attendees/summit). Integrated into the summits will be focus groups, each including ~ 5–10 participants for a total of ~ 10–15 focus groups (5 per summit).

#### Data collection

Reports will be shared with summit participants 2 weeks ahead of the summit and then presented to and discussed with participants at the summit. Duration of each summit will be 3 h. An experienced facilitator with strong skills in working with heterogeneous groups (including immigrants, racialized, and low-income individuals, and those living with dementia) and mitigating power differences will facilitate summits. Summits will follow our previously tested, theory-based, effective process of feeding back and discussing study findings [104–109].

In the first half of the summit, we will present and discuss QoL reports. Sessions will be recorded for data analyses (transcription purposes), using Zoom. We will then assign participants by role to focus groups (60–90 min, 5–10 people each). We will conduct separate focus groups with 1) decision makers, 2) NH managers, 3) nurses and other regulated care staff, 4) care aides, and 5) advocates (e.g., Alzheimer Societies), individuals in need of care and their family/friend caregivers. Each focus group will be facilitated by a research team member and video recorded (for transcription purposes). Focus groups following large panel discussions will create space for individual knowledge users to share their experiences and perspectives in detail and to raise issues that they may have decided not to raise in the large group.

#### Data analyses

We will transcribe summit and focus group recordings verbatim. Using thematic content analysis [110], we will deductively code transcripts, focusing on participants’ interpretations of results, perceived usefulness and intended uses of our data, promising practices, and new strategies to maximize QoL and minimize QoL inequities. Using the constant comparative method [111], we will inductively identify additional themes not captured by our focus group guide (Additional file 5). For rigour and trustworthiness, each of 2 team members will independently code texts and then reconcile coding together until consensus is reached.

## Discussion

QoL and its social determinants are essential aspects largely missing from routine data collected in NHs. US research has assessed disparities in NH care in general [34], and especially racial disparities in resident QoL [29–33]. However, we lack studies assessing multiple social determinants (and their complex interplay – their intersection) and their association with resident QoL directly (rather than focusing on quality of care or healthcare access). This study will contribute to addressing these important knowledge gaps. Our findings will create the prerequisites for developing and testing tailored strategies in future studies aimed at measuring and improving QoL for NH residents living with dementia and reducing inequities in QoL. Our intersectional approach will be a particular strength in informing complex improvement interventions. Our approach to QoL assessment provides a baseline assessment of QoL across several provinces in Canada. Used repeatedly, it can advance monitoring of the impact of diverse strategies and policy initiatives for QoL, improving their resource allocation. This study provides the foundational methods and data for evaluative work to investigate in depth the mechanisms leading to QoL inequities in NHs, and for longitudinal studies to identify trajectories in QoL. Our advanced skill-building for trainees will develop urgently needed Canadian capacity in research on QoL and its social determinants in vulnerable populations living with dementia. Our CIHR-funded project [112] will contribute to the science of sustaining, spreading, and scaling successful implementation of tailored strategies to improve QoL and reduce inequities in NHs. These learnings will be key to longitudinal work and to expanding our QoL work to other Canadian provinces and territories.

### Supplementary Information

**Additional file 1.** DEMQOL-CH.

**Additional file 2.** Nursing home resident social determinants.

**Additional file 3.** Care aide demographics.

**Additional file 4.** Nursing home characteristics.

**Additional file 5.** Focus group guide – feedback summit participants.

## Data Availability

All study data and analytical output will be held on a secure server at the University of Alberta, as required by the ethics and provincial data confidentiality requirements relevant to this study. As such, all study datasets will not be publicly available. Ethics approvals and provincial data confidentiality regulations require that access will be restricted to approved study investigators and research associates. Aggregate data and relevant statistical code will be available from the primary investigator (MH) on reasonable request. For additional information regarding the availability of data contact Dr. Matthias Hoben, email: mhoben@yorku.ca.

## Abbreviations

LCA: Latent class analyses
NH: Nursing home
QoL: Quality of life

## Footnotes

1 We base our understanding of the term ‘racialization’ on Hochman’s [113] comprehensive discussion and defence of this term. Specifically, racialization is defined “as the process through which groups come to be understood as major biological entities and human lineages, formed due to reproductive isolation, in which membership is transmitted through biological descent.” [113], p. 1246. The terminology related to race and ethnicity is highly heterogeneous and contested, and even the authors of the US studies cited above seem to vary the terminology they use from paper to paper [30–34]. In this paper, we refer to racialized residents as those who are perceived and grouped by others based on their appearance, behaviours, immigration background, ‘foreign’ accent, etc., and who often experience discrimination and inequities because of that.
