# Supplementary material for: Counting what counts: assessing quality of life and its social determinants among nursing home residents with dementia
Source: BMC Geriatr. 2024 Feb 21;24:177. doi: 10.1186/s12877-024-04710-1 (PMC10880372; doi:10.1186/s12877-024-04710-1)
Supplement: Supplementary file 1 — Additional file 1. DEMQOL-CH. [file 12877_2024_4710_MOESM1_ESM.pdf]

DEMQOL-CH - Instructions for administration

Aim of the questionnaire

- It is the resident/service user's feelings and understandings that are of interest. There are no right or wrong answers
- How people feel about things that happen every day are important. Things such as the activities that people do during the day, how they feel, and their relationships.
- Do not answer questions in terms of ability/function, it is about how much the resident/service user worries about the activities.
- **<u>You will need a DEMQOL-CH questionnaire for each resident</u>**

Completing the questionnaire

- Read the instructions on the front of the questionnaire.
- Read and complete the practice question.
- Read each question exactly as it is written
- Mark only one response for each question
- At the end, go back to any missed items
- **<u>Complete each questionnaire by yourself, do not ask other members of staff or the resident/service user.</u>**

Possible queries

I don't understand a question

If you are struggling with the meaning of the question re-read the question again, including the stem and item. Base your answer on what you think the question means.

I don't know the answer or I am unsure of an answer

It can be hard to choose a response, choose the response option that most applies to the resident/service user. All the questions are very important, please try to answer all of the questions.

Can I get the resident/service user to complete it with me?

Understanding the feelings of people who live in care and nursing homes is the aim questionnaire. Some people may not be able to provide this information themselves because of memory or other impairments. Therefore, getting care staff opinions is the best way to obtain a consistent measurement of how residents/service users feel.

I am unsure how the resident/service user is 'feeling'

You probably know the resident quite well, just give the answer that best describes how you think the resident has felt.

I don't understand the general quality of life questionnaire at the end

Carefully re-read the stem and the question again. Think of how the resident/service user would rate their quality of life overall, thinking about the resident/service user's life in the last week, would you say it was . . . very good, good, fair, or poor?

DEMQOL - CH

<u>Instructions</u>

I would like to ask you about the resident's life, as you are the person who knows him/her best. There are no right or wrong answers. Just give the answer that best describes how the resident has felt in the last week. If possible try and give the answer that you think the resident would give. Don't worry if some questions appear not to apply to the resident. We have to ask the same questions of everybody.

For all of the questions I'm going to ask you, I want you to think about the last week.

First I'm going to ask you about ________________*(the resident's)* **<u>feelings</u>. In the last week:**

1.	**would you say that ______has felt cheerful? ****	☐ a lot	☐ quite a bit	☐ a little	☐ not at all
2.	**would you say that ______has felt worried or anxious?**	☐ a lot	☐ quite a bit	☐ a little	☐ not at all
3.	**would you say that ______ has felt frustrated?**	☐ a lot	☐ quite a bit	☐ a little	☐ not at all
4.	**would you say that ______ has felt full of energy? ****	☐ a lot	☐ quite a bit	☐ a little	☐ not at all
5.	**would you say that ______ has felt sad?**	☐ a lot	☐ quite a bit	☐ a little	☐ not at all
6.	**would you say that ______has felt content? ****	☐ a lot	☐ quite a bit	☐ a little	☐ not at all
7.	**would you say that ______ has felt distressed?**	☐ a lot	☐ quite a bit	☐ a little	☐ not at all
8.	**would you say that ______ has felt lively? ****	☐ a lot	☐ quite a bit	☐ a little	☐ not at all
9.	**would you say that ______ has felt irritable?**	☐ a lot	☐ quite a bit	☐ a little	☐ not at all
10.	**would you say that ______ has felt fed-up?**	☐ a lot	☐ quite a bit	☐ a little	☐ not at all
11.	**would you say that ______ has felt that he/she has things to look forward to? ****	☐ a lot	☐ quite a bit	☐ a little	☐ not at all

Next, I'm going to ask you about ________________*(the resident's)* **<u>memory</u>. In the last week:**

12.	**how <u>worried</u> would you say _______ has been about his/her memory in general?**	☐ a lot	☐ quite a bit	☐ a little	☐ not at all
13.	**how <u>worried</u> would you say _______ has been about forgetting things that happened a long time ago?**	☐ a lot	☐ quite a bit	☐ a little	☐ not at all

14.	**how <u>worried</u> would you say _______ has been about forgetting things that happened recently?**	☐ a lot	☐ quite a bit	☐ a little	☐ not at all
15.	**how <u>worried</u> would you say _______ has been about forgetting people's names?**	☐ a lot	☐ quite a bit	☐ a little	☐ not at all
16.	**how <u>worried</u> would you say _______ has been about forgetting where he/she is?**	☐ a lot	☐ quite a bit	☐ a little	☐ not at all
17.	**how <u>worried</u> would you say _______ has been about forgetting what day it is?**	☐ a lot	☐ quite a bit	☐ a little	☐ not at all
18.	**how <u>worried</u> would you say _______ has been about his/her thoughts being muddled?**	☐ a lot	☐ quite a bit	☐ a little	☐ not at all
19.	**how <u>worried</u> would you say _______ has been about difficulty making decisions?**	☐ a lot	☐ quite a bit	☐ a little	☐ not at all
20.	**how <u>worried</u> would you say _______ has been about making him/herself understood?**	☐ a lot	☐ quite a bit	☐ a little	☐ not at all

Now, I'm going to ask about _________*(the resident's)* <u>everyday life</u>. In the last week

21.	**how <u>worried</u> would you say _______ has been about keeping him/herself clean (e.g. washing and bathing)?**	☐ a lot	☐ quite a bit	☐ a little	☐ not at all
22.	**how <u>worried</u> would you say _______ has been about keeping him/herself looking nice?**	☐ a lot	☐ quite a bit	☐ a little	☐ not at all
23.	**how <u>worried</u> would you say _______ has been about getting what he/she wants from the shops?**	☐ a lot	☐ quite a bit	☐ a little	☐ not at all
24.	**how <u>worried</u> would you say _______ has been about using money to pay for things?**	☐ a lot	☐ quite a bit	☐ a little	☐ not at all

25.	**how <u>worried</u> would you say _______ has been about looking after his/her finances?**	☐ a lot	☐ quite a bit	☐ a little	☐ not at all
26.	**how <u>worried</u> would you say _______ has been about things taking longer than they used to?**	☐ a lot	☐ quite a bit	☐ a little	☐ not at all
27.	**how <u>worried</u> would you say _______ has been about getting in touch with people?**	☐ a lot	☐ quite a bit	☐ a little	☐ not at all
28.	**how <u>worried</u> would you say _______ has been about not having enough company?**	☐ a lot	☐ quite a bit	☐ a little	☐ not at all
29.	**how <u>worried</u> would you say _______ has been about not being able to help other people?**	☐ a lot	☐ quite a bit	☐ a little	☐ not at all
30.	**how <u>worried</u> would you say _______ has been about not playing a useful part in things?**	☐ a lot	☐ quite a bit	☐ a little	☐ not at all
31.	**how <u>worried</u> would you say _______ has been about his/her physical health?**	☐ a lot	☐ quite a bit	☐ a little	☐ not at all

We've already talked about lots of things: ________________(the resident's)_ **feelings, memory and everyday life. Thinking about all of these things in the last week:**

32.	**how would you say _________ would rate his/her quality of life overall? ****	☐ very good	☐ good	☐ fair	☐ poor

****** items that need to be reversed before scoring

Please answer the question below

HOW CONFIDENT DID YOU FEEL IN COMPLETING THE QUESTIONNAIRE?

Not confident at all	Not very confident	Neither confident nor unconfident	Confident	Very confident
1	2	3	4	5
