# Supplementary material for: Counting what counts: assessing quality of life and its social determinants among nursing home residents with dementia
Source: BMC Geriatr. 2024 Feb 21;24:177. doi: 10.1186/s12877-024-04710-1 (PMC10880372; doi:10.1186/s12877-024-04710-1)
Supplement: Supplementary file 2 — Additional file 2. Nursing home resident social determinants. [file 12877_2024_4710_MOESM2_ESM.pdf]

## Social determinants survey – Counting What Counts Study

### Instructions for completion

This form is completed by the study coordinator or research assistant (as an electronic version). The study coordinator/research assistant works with care teams to obtain the required information. Care teams are encouraged to talk to the resident themselves, to the resident's family/friend care partners and to colleagues.

### Resident Identification

Resident ID: ________________________________________________________________

### Resident Information

a. What is this person's gender?
   *Refers to current gender which may be different from sex assigned at birth and may be different from what is indicated on legal documents.*
   ❑ Male
   ❑ Female
   ❑ Or please specify this person's gender ________________
b. What is this person's religion?
   Indicate a specific denomination or religion even if this person is not currently a practising member of that group.
   For example, Roman Catholic, United Church, Anglican, Muslim, Baptist, Hindu, Pentecostal, Lutheran, Presbyterian, Sikh, Buddhist, Jewish, Greek Orthodox, etc.
   For additional examples of denominations and religions, visit www12.statcan.gc.ca/religion-e
   ❑ Specify one denomination or religion only _____________
   or
   ❑ No religion
c. What is the highest certificate, diploma or degree that this person has completed?
   ❑ Less than high school diploma or its equivalent
   ❑ High school diploma or a high school equivalency certificate
   ❑ Trade certificate or diploma
   ❑ College, CEGEP or other non-university certificate or diploma (other than trades certificates or diplomas)
   ❑ University certificate or diploma below the bachelor's level
   ❑ Bachelor's degree (e.g. B.A., B.Sc., LL.B.)
   ❑ University certificate, diploma or degree above the bachelor's level
d. What is this person's sexual orientation
   ❑ Heterosexual (sexual relations with people of the opposite sex)
   ❑ Homosexual, that is lesbian or gay (sexual relations with people of your own sex)
   ❑ Bisexual (sexual relations with people of both sexes)
e. Are LTC costs of this person fully publicly covered? ❑ Yes ❑ No

**Ethnocultural background**

f. Please select which one or more racial or cultural groups on the following list this person belongs to.
- ❑ White
- ❑ South Asian (e.g., East Indian, Pakistani, Sri Lankan)
- ❑ Chinese
- ❑ Black
- ❑ Filipino
- ❑ Latin American
- ❑ Arab
- ❑ Southeast Asian (e.g., Vietnamese, Cambodian, Malaysian, Laotian)
- ❑ West Asian (e.g., Iranian, Afghan)
- ❑ Korean
- ❑ Japanese
- ❑ Other – Specify _________________

g. Is this person or has this person ever been a landed immigrant in Canada?
- ❑ Yes (go to g)
- ❑ No

*h.* In what year did this person first become a landed immigrant in Canada? ________
*If respondent cannot give exact year of immigration, ask for a best estimate of the year*

i. What is the language that this person first learned at home in childhood and still understands?
- ❑ English
- ❑ French
- ❑ Other – Specify _________________

**Social Support**

j. How many children does this person have? _____

k. How many family members or friends visit the resident regularly? _____

l. Does this person have a legal guardian? ❑ Yes ❑ No
