# Supplementary material for: Counting what counts: assessing quality of life and its social determinants among nursing home residents with dementia
Source: BMC Geriatr. 2024 Feb 21;24:177. doi: 10.1186/s12877-024-04710-1 (PMC10880372; doi:10.1186/s12877-024-04710-1)
Supplement: Supplementary file 3 — Additional file 3. Care aide demographics. [file 12877_2024_4710_MOESM3_ESM.pdf]

Demographic survey – Counting What Counts study

1. Please indicate your **highest** educational level (only select one option):

☐1 High school degree

☐2 Diploma/Certificate

☐3 Bachelors Degree

☐4 Masters Degree

☐5 PhD/PharmD

2. Is English your first language?

☐1 Yes

☐2 No – please specify your first language:___________

3. What is your **primary** role in the facility (select only one)?

☐1 Health Care Aide, Personal Care Attendant, Special Care Aide, Nursing Attendant

☐2 Licensed Practical Nurse

☐2 Clinical educator/specialist

☐3 RAI coordinator

☐4 Care manager

☐5 Director of care

☐5 Facility administrator

☐5 Medical director

☐5 Other (please specify) ____

4. Please indicate your age range according to the following groups:

☐1 ≤ 30 years

☐2 31-40years

☐3 41-50 years

☐4 51-60years

☐5 > 60 years

5. What best describes your gender?

☐1 Woman

☐2 Man

☐3 Prefer to self-describe: __________

☐4 Prefer not to answer

6. How long have you worked in your current position?

[ ] Years [ ] Months

7. You may belong to one or more racial or cultural groups on the following list. Please select all that apply. Are you…

☐1 White
☐2 South Asian (e.g. East Indian, Pakistani, Sri Lankan)
☐3 Chinese
☐4 Black
☐5 Filipino
☐6 Latin American
☐7 Arab
☐8 Southeast Asian (e.g. Vietnamese, Cambodian, Malaysian, Laotian)
☐9 West Asian (e.g. Iranian, Afghan)
☐10 Korean
☐11 Japanese
☐12 Other – please specify:

8. Pick the answer that indicates how often, if ever, you have experienced the following feelings. If you have never experienced this thought or feeling, select "never". If you did have this thought or feeling, select the best fitting answer.

Answer these questions in general, that is not just about [name of unit].

	Never	A Few Times a Year or Less	Once a Month or Less	A Few Times a Month	Once a Week	A Few Times a Week	Daily
1. I feel tired when I get up in the morning and have to face another day on the job.	☐0	☐1	☐2	☐3	☐4	☐5	☐6
2. In my opinion, I am good at my job.	☐0	☐1	☐2	☐3	☐4	☐5	☐6
3. I just want to do my job and not be bothered.	☐0	☐1	☐2	☐3	☐4	☐5	☐6

4. Working all day is really a strain for me.	☐0	☐1	☐2	☐3	☐4	☐5	☐6
5. I have become more cynical about whether my work contributes anything.	☐0	☐1	☐2	☐3	☐4	☐5	☐6
6. I feel exhilarated when I accomplish something at work.	☐0	☐1	☐2	☐3	☐4	☐5	☐6
7. I have become less enthusiastic about my work.	☐0	☐1	☐2	☐3	☐4	☐5	☐6
8. I feel burned out from my work.	☐0	☐1	☐2	☐3	☐4	☐5	☐6
9. I have accomplished many worthwhile things in this job.	☐0	☐1	☐2	☐3	☐4	☐5	☐6

MBI-General Survey: Copyright ©1996 Wilmar B. Schaufeli, Michael P. Leiter, Christina Maslach & Susan E. Jackson.
All rights reserved in all media. Published by Mind Garden, Inc., www.mindgarden.com
