# Supplementary material for: Counting what counts: assessing quality of life and its social determinants among nursing home residents with dementia
Source: BMC Geriatr. 2024 Feb 21;24:177. doi: 10.1186/s12877-024-04710-1 (PMC10880372; doi:10.1186/s12877-024-04710-1)
Supplement: Supplementary file 4 — Additional file 4. Nursing home characteristics. [file 12877_2024_4710_MOESM4_ESM.pdf]

Facility survey – Counting What Counts Study

Instructions for completion

This form is completed by the study coordinator (as an electronic version). The study coordinator works with the appropriate persons in the facility to obtain the required information. These persons usually are the facility administrator, director of care, unit managers, quality improvement specialists, clinical educators, etc.

Facility Identification

Facility ID: ____

Facility Information

a. Please select one of the following: The owner – operator model for this facility is:
   - ❑ public not for profit
   - ❑ voluntary not for profit
   - ❑ private for profit

b. Please select whether the site is located in an urban or rural area
   - ❑ Urban (i.e. located within a population centre with a population of at least 1,000)
   - ❑ Rural (i.e. not located within a population centre)

   How many beds does your facility have? ____

c. How many care units are in your facility? ____

d. Has the facility implemented a person-centred model of care?
   - ❑ yes – the Butterfly Household Model of Care
   - ❑ yes – the Eden Alternative® model
   - ❑ yes – the Green House Model
   - ❑ yes – any other model (specify: __________________)
   - ❑ No

e. What proportion of residents living in the facility are members of a visible minority? ____
   (Visible minority refers to whether a person belongs to a visible minority group as defined by the Employment Equity Act and, if so, the visible minority group to which the person belongs. The Employment Equity Act defines visible minorities as "persons, other than Aboriginal peoples, who are non-Caucasian in race or non-white in colour." The visible minority population consists mainly of the following groups: South Asian, Chinese, Black, Filipino, Latin American, Arab, Southeast Asian, West Asian, Korean and Japanese.)

Staffing

Number of **RNs** on average that are scheduled daily in the facility:

	Monday-Friday	Saturday	Sunday
**Days**			
**Evenings**			
**Nights**			
**Other 1**<br>**- specify name ________**<br>**- specify length _________**			
**Other 2**<br>**- specify name _________**<br>**- specify length _________**			

Number of **LPNs** on average that are scheduled daily in the facility:

	Monday-Friday	Saturday	Sunday
**Days**			
**Evenings**			
**Nights**			
**Other 1**<br>**- specify name ________**<br>**- specify length _________**			
**Other 2**<br>**- specify name _________**<br>**- specify length _________**			

Number of **care aides** on average that are scheduled daily in the facility:

	Monday-Friday	Saturday	Sunday
**Days**			
**Evenings**			
**Nights**			
**Other 1**<br>**- specify name ________**<br>**- specify length _________**			
**Other 2**<br>**- specify name _________**<br>**- specify length _________**			
