# Supplementary material for: Counting what counts: assessing quality of life and its social determinants among nursing home residents with dementia
Source: BMC Geriatr. 2024 Feb 21;24:177. doi: 10.1186/s12877-024-04710-1 (PMC10880372; doi:10.1186/s12877-024-04710-1)
Supplement: Supplementary file 5 — Additional file 5. Focus group guide – feedback summit participants. [file 12877_2024_4710_MOESM5_ESM.pdf]

Focus group guide – feedback summit participants

Preamble

Thank you for participating in this focus group. We are interviewing you because you are a participant in our feedback summit on quality of life (QoL) and its health and social determinants. Your feedback will help us to assess how well our findings resonate with your experience, how you interpret these findings, how useful you find the QoL data reported back to you, and whether and how you plan to use these data in your own practice.

Focus Group Question Guide

Instructions to the interviewer:

- There are main *numbered* questions. Try to proceed through them in the order they appear, but also give people the freedom to move beyond the boundaries of each question to some extent.
- You can also ask additional ***probing*** questions as a follow up to things you hear if you think this will provide additional insights.
- The key to good focus group facilitation is picking up on and probing when relevant comments are made (saying things like "tell me more about that") and steering people back on track when they get off topic.

Questions and probes:

1. Overall, of the results we just presented on and of the subsequent discussions, was there anything that stood out to you or that you want to discuss in more detail?
2. As a [add role of knowledge user, i.e. decision makers, LTC managers, person working in LTC, person in need of care or their family/friend care partner or advocate] what do our findings on QoL and its health and social determinants mean to you?
    a. Anything that confirms or contradicts your experience?
    b. Anything surprising?
    c. How do you explain these findings?
3. What do you think are the main reasons for poor QoL in LTC and what do you think are the main reasons for QoL inequities in LTC?
4. For decision makers: what is your province/health region already doing to maximize LTC resident QoL and to minimize QoL inequities? Are you aware of any promising practices? What do you think can/should be done differently?
5. For LTC managers and staff: what is your organization already doing to maximize LTC resident QoL and to minimize QoL inequities? Are you aware of any promising practices? What do you think can/should be done differently?
6. For citizens and advocates: what are LTC systems and organizations already doing to maximize LTC resident QoL and to minimize QoL inequities? Are you aware of any promising practices? What should be done differently?

Thank you very much for participating in this focus group!
